# An intricate role of Ang II/AT1 in the modulation of monosodium glutamate-induced pulmonary fibrosis by TGF-β/Smad through quercetin

**DOI:** 10.1038/s41598-025-05781-9

**Published:** 2025-07-01

**Authors:** Arnab Banerjee, Debasmita Das, Krishnendu Manna, Jayati Roy, Sandip Mukherjee, Bithin Kumar Maji

**Affiliations:** 1 Department of Physiology (UG & PG), Serampore College, 9 William Carey Road, Hooghly, Serampore, 712201 West Bengal India; 2 Department of Food and Nutrition, University of Kalyani, Nadia, Kalyani, West Bengal India

**Keywords:** Quercetin, Monosodium glutamate, Pulmonary fibrosis, Pro-fibrotic cytokines, Oxidative stress, TGF-β/Smad, Biochemistry, Molecular biology, Physiology, Health care, Molecular medicine

## Abstract

To investigate the protective actions of the natural flavonoid quercetin against monosodium glutamate (MSG)-induced pulmonary fibrosis in rats, the present study targets the modulation of the TGF-β/Smad signaling pathway and the involvement of Ang II/AT1. The experimental model involved the treatment of rats with MSG (0.6 g/kg body weight) for 4 weeks and quercetin dosages of 25 mg, 50 mg, and 100 mg/kg body weight. The study applied the combination of biochemical, molecular, and histopathological evaluation to identify the role of quercetin in impacting major cytokines (IL-17, IL-19, TGF-β, VEGF), oxidative stress markers (TBARS, NO, SOD, CAT, GSH), extracellular matrix components (collagen-I, α-SMA, fibronectin), and fibrosis gene expression (TGF-β1, Smad2/3/4, CTGF, Snail, Slug). MSG treatment increased pro-fibrotic cytokines, oxidative stress, and deposition of collagen in a significant amount, while administration of quercetin dose-dependently reversed the alterations. Quercetin also reversed the activity of antioxidant enzymes, reduced inflammatory cytokines, and inhibited TGF-β/Smad signaling as indicated by lowered TGF-β receptor II activation and following Smad phosphorylation. Molecular docking demonstrated that quercetin competitively binds to TGF-β receptor II to inhibit MSG-induced fibrotic signaling. Quercetin inhibits MSG-induced lung fibrosis by inhibiting collagen accumulation and inflammatory cell invasion and has the potential to produce therapeutic effects by modulating TGF-β/Smad signaling and restoring lung tissue homeostasis.

**Supplementary Information:**

The online version contains supplementary material available at 10.1038/s41598-025-05781-9.

## Introduction

Pulmonary fibrosis is a chronic lung disease involving excessive accumulation of extracellular matrix components, especially collagen, resulting in tissue remodeling and compromised respiratory function^1^. This pathological process is initiated by dysregulated cellular signaling pathways, such as the activation of important cytokines and growth factors like TGF-β, IL-17, IL-19, and VEGF that enhance inflammatory processes, collagen deposition, and angiogenesis^2–7^. Hypoxic conditions additionally amplify these mechanisms by upregulating hypoxia-inducible factor (HIF)-1α, disturbing cellular homeostasis, and producing an imbalance of pro-fibrotic versus anti-fibrotic signals^8,9^. Additionally, the downregulation of such protective factors as pigment epithelium-derived factor (PEDF) and endostatin plays a role in the development of fibrotic phenotype^10,11^. Despite progress in the comprehension of the molecular basis of pulmonary fibrosis, therapeutic interventions are limited, highlighting the need for new treatment approaches.

Monosodium glutamate (MSG) is one of the most common flavoring agents known to induce adverse health effects, especially in the development of lung pathology^12,13^. MSG induces the generation of reactive oxygen species to disturb the redox-guided physiological homeostasis; furthermore, it can also activate different pro-inflammatory factors like IL-6, TNF-α, NF-kB, and pro-fibrotic TGF-β, and VEGF to cause serious necrotic changes in the rat model^14–17^. Its role in pulmonary fibrosis has recently received much attention in this area. Accumulating evidence suggested that activation of pro-inflammatory and pro-fibrotic cytokines by a xenobiotic substance promotes pulmonary fibrosis by hypoxia-mediated processes^2–7^.

Considering the failure of specific therapies for MSG-induced pulmonary anomaly, there is a promising interest in investigating natural compounds with antioxidant, anti-fibrotic, and anti-inflammatory actions as therapeutic drugs. Therefore, the present study hypothesizes that quercetin, a natural flavonoid with documented antioxidant and anti-inflammatory properties^17,18 ^could be a viable modulator against MSG-induced lung fibrosis. The present study raised the question of whether quercetin inhibits MSG-induced pulmonary fibrosis in rats through modulating the cellular signaling. Hence, the aim of the present work mainly focused on the key cytokines and growth factors in mediating pulmonary alterations to promote the disruptions of physiological homeostasis by activating TGF-β/Smad, mediated by MSG, and the possible protective effects of quercetin against restoring homeostatic balance in these pathological mechanisms.

## Methods

### Chemicals and reagents

Quercetin (SRL: 71923) and monosodium glutamate (SRL: 23229) were purchased from SRL, India. The chemicals and reagents used in the study were of high grade and procured from Merck (Germany) and Sigma Aldrich (USA). Gibco (USA) provided the cell culture medium, buffer, and all other reagents.

### Preparation of the treatment and supplementary substance

Freshly prepared MSG was dissolved in distilled water and force-fed to the rat at a dose of 0.6 g/kg of body weight for 4 weeks via a gavage needle^14,17^. On the other hand, quercetin was also dissolved in distilled water and administered orally using a gavage needle in continuation of the treatment period with or without MSG in dosages of 25 mg/kg, 50 mg/kg, and 100 mg/kg of body weight^17^. In addition, earlier work uncovered that quercetin was nontoxic at a dose of 2,000 mg/kg body weight via the oral route, thus implying that the oral LD_50_ of quercetin in rats was more than 2000 mg/kg of body weight^17,19^. Since there are no reported adverse effects of quercetin in animal or human studies, quercetin is categorized as “generally recognized as safe“^20^.

### Experimental design

**Fig. 1:**
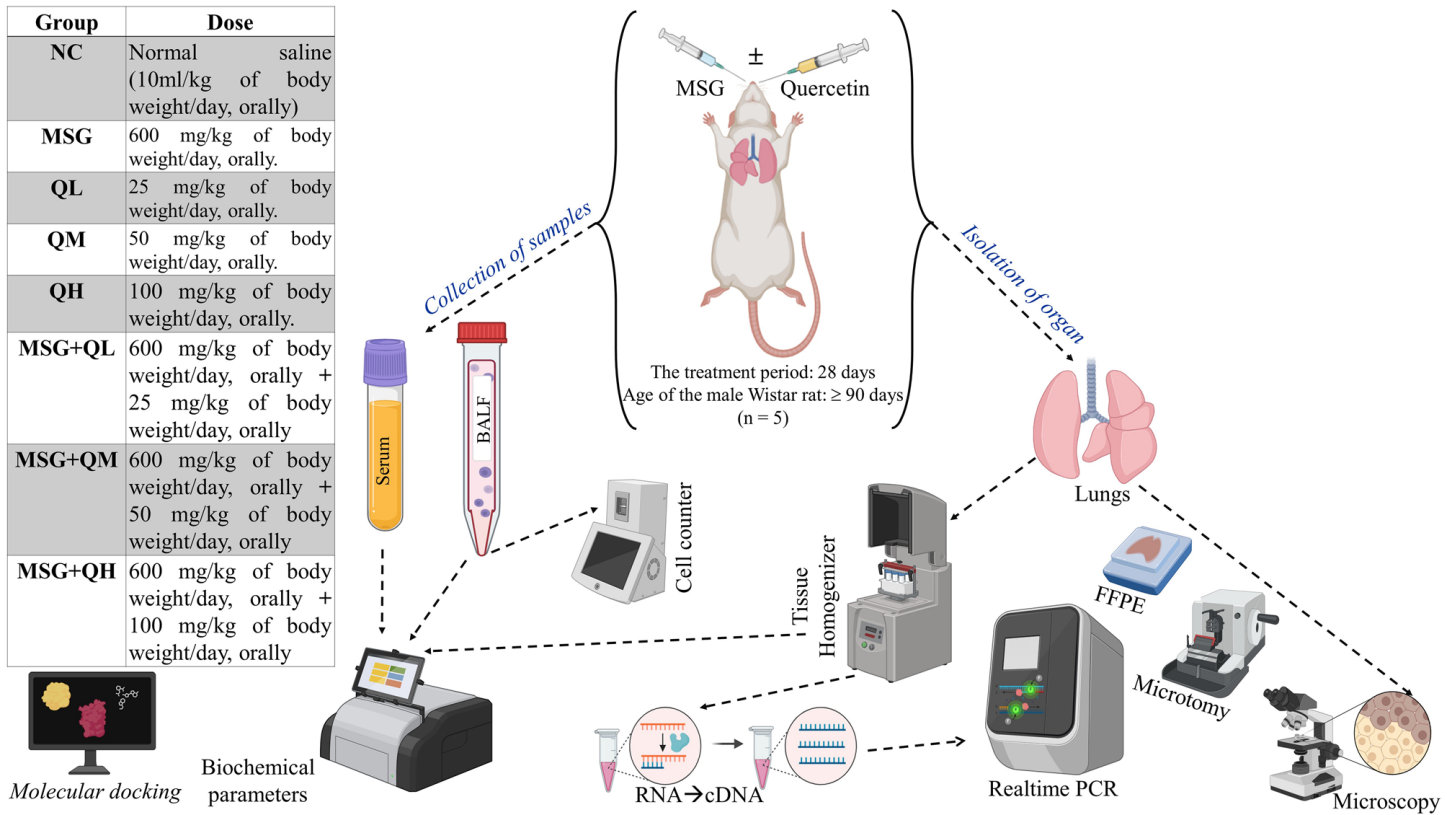
Experimental design (BioRender was used to make the figure: https://biorender.com).

Eight equal groups of adult male Wistar rats (Age: ≥90 days, *n* = 5) were randomly selected, divided, and administered the treatments shown in Fig. 1. All rats were kept in their cages with a 12-hour day-night cycle with artificial lighting and ≥12 times/hour, the barrier system animal room conditions were controlled at 22 ± 2°C and relative humidity of 40–70%. The study did not employ human subjects. The animals had free access to drinking water and nutritious granular feed. All rats were given a control diet prepared with 71% carbohydrates, 18% protein, 7% fat, and 4% salt mixture and water ad libitum. After the treatment period, animals from different groups that fasted overnight were administered intraperitoneal injections (IP) of ketamine at a dose of 87 mg/kg and xylazine at a dose of 13 mg/kg of body weight, as recommended by the Institutional Animal Ethics Committee (IAEC), Serampore College. This was done to collect blood, prepare serum, and isolate lungs for measuring different parameters^14,17^. The experiment protocols in this research were subject to review and received clearance from the IAEC of Serampore College, with the given approval number 25/P/S/SC/IAEC/2019, registered under (1946/PO/Re/S/17/CPCSEA), the Committee for Control and Supervision of Experiments on Animals (CCSEA). All experiments were performed according to the relevant guidelines and regulations of the IAEC, Serampore College. Moreover, all the animal experiments were carried out per the Animal Research: Reporting of In Vivo Experiments (ARRIVE) guidelines.

### Measurement of cytokines and Ang II in serum

Rat standard ELISA kits were used to determine different cytokine levels by using a microtiter plate reader; such as interleukin (IL)-17 from CUSABIO (USA), IL-19 from MyBioSource (CA, USA), vascular endothelial growth factor (VEGF) from RayBiotech (USA), transforming growth factor (TGF)-β from eBioscience (USA), HIF-1α, platelet-derived growth factor (PDGF), PEDF, endostatin from Shanghai Lanji Biotechnology Co., Ltd. (China), laminin (LN), high mobility group box 1 (HMGB1), hyaluronic acid (HA) from Shanghai yuanye Bio-Technology Co., Ltd. (China) and angiotensin (Ang) II from CUSABIO (USA) respectively were estimated by the instruction manual of the kits.

### Isolation of bronchoalveolar lavage fluid (BALF) followed by the total and differential cell counts

The thoracic cavity was opened to obtain BALF; the trachea was exposed and connected to a 16-gauge catheter. Mammalian saline solution was administered into the right lung through the cannula as the left major bronchi were occluded. The sample mixed with saline was centrifuged at 4°C and 3500 rpm for 10 min. To determine the differential and total cell counts, precipitated cells were harvested and resuspended in 0.5 mL saline solution. BALF supernatant was obtained for further investigation. Total and differential cell counts in BALF were identified and determined by using Wright-Giemsa staining. The BALF was centrifuged, and the cell pellets were suspended in 0.5 ml of normal saline. After that, the slides were stained with Wright-Giemsa staining for 10 min. Differential cell count was performed by taking the count of neutrophils and lymphocytes on the slides at 40x magnification by counting the total of 200 cells per slide, the counts were displayed as the number of cells per ml^21^.

### Measuring the concentration of total proteins, lactate dehydrogenase (LDH), TGF-β, and HIF-1α levels in BALF

The total protein content was estimated using a kit (Spinreact Co., Girona, Spain) by the manufacturer’s protocol, and the absorbance was estimated at 598 nm. LDH activity was measured using a commercial kit (Abcam, UK). Change in absorbance was measured at 340 nm and represented in U/L. Furthermore, the standard ELISA kits also estimated TGF-β (CUSABIO, USA) and HIF-1α (Bioassay Technology Laboratory Company, China) levels in BALF.

### Preparation of lung tissue extract

Lung tissue homogenates were prepared with a protease inhibitor cocktail with PBS^14,22^. It has been prepared to determine the level of free radicals, nonenzymatic and enzymatic antioxidants markers [e.g, nitric oxide (NO) and thiobarbituric acid-reactive substances (TBARS), superoxide dismutase (SOD), glutathione (GSH), and catalase (CAT)] of oxidative stress and other inflammatory parameters.

### Estimation of free radicals and antioxidants

Free radicals such as NO and TBARS were quantified using the Griess reaction^23^ and the TBA assay^24^. The level of antioxidants such as SOD, CAT, and GSH were determined by the NBT method^25,26^and Elman’s method^27^.

### Estimation of TGF-β, fibronectin, collagen-I (Col-I), α-SMA, E-cadherin, vimentin, and N-cadherin content in lung tissues

The standard ELISA kits were used in the quantitative ELISA to estimate the levels of TGF-β, N-Cadherin, fibronectin, vimentin (MyBioSource, CA, USA), Col-I, α-SMA (CUSABIO, USA), and E-Cadherin (Abcam, UK) in lung tissue homogenate.

### Determination of hydroxyproline and total nitrite/nitrate (NOx) content in lung tissues

Lung hydroxyproline content was quantified in lung tissues by initiating the procedure with overnight incubation of lung specimens in a 5% KOH solution at 37°C, facilitating alkaline-mediated hydrolysis. After this incubation, tissues were hydrolyzed with 10N NaOH, and the chloramine-T solution was added for 3 h at room temperature for a colorimetric product formation. These samples were mixed with Ehrlich’s reagent and kept in a water bath at 65°C for 20 min. This allowed the generation of a chromophore detectable at λ = 550 nm. Therefore, hydroxyproline content was estimated and expressed in µg/of tissue^21^.

On the other hand, the content of NOx was assayed by a method already standardized by a group of researchers^28^. The reduction of nitrate by vanadium (III) followed by detection with acidic Griess reagent, is the main principle of the method. The absorbance of the colored complex was measured at 540 nm by using a spectrophotometer. To find the NOx content in µmol/g of tissue for each of the samples, data points were interpolated from the standard curve obtained in parallel, which assured the quantification of nitrite and nitrate levels.

### Estimation of thioredoxin-interacting protein (TXNIP) and HIF-1α contents in lung tissues

TXNIP and HIF-1α were estimated in lung tissues from different groups of animals utilizing standard ELISA kits (Bioassay Technology Laboratory, Shanghai, China).

### Histological analysis

Hematoxylin and eosin (HE), picrosirius red (PSR), and Masson’s Trichrome (MT) staining techniques were alternatively used to stain the lung tissue samples. A camera-equipped compound microscope (Primo Star, Carl Zeiss Meditec, Dublin, CA) was used for analysing stained microscopic slides.

### Quantitative real-time PCR (qRT-PCR)

β-actin was used as an internal control for AT1, CTGF, Snai1, Slug, TGF-β1, TGF-β receptor (TβR) I, TβR II, Smad 2, Smad 3, Smad 4, IL-6, IL-1β, TNF-α, HMGB1, MMP-2, MMP-9, Fibronectin, Col1a1, Col1a2, Col3a1, and α-SMA (Supplementary material 1). Trizol reagent was used to extract the RNA from lung tissues as per the manufacturer’s protocol (Invitrogen, Carlsbad, CA, USA). The high-capacity cDNA reverse transcription kit of Applied Biosystems (Foster City, CA) was used to reverse-transcribe the RNA into cDNA. Amplification of target genes was performed with the Invitrogen SYBR Green Real-time PCR Master Mixes, using the technique of qRT-PCR^17^.

### Molecular Docking

AutoDock Vina (v1.1.2) was applied in the molecular docking experiments to elucidate the interaction between MSG, quercetin, and TβR-II. Ligands were derived from PubChem, whereas the structure of the receptor was downloaded from the protein data bank (PDB). In receptor preparation, polar hydrogens are added and then optimized by the use of AutoDock Tools (ADT), while charges to the ligand have been assigned as Gasteiger. A docking grid of 40 Å × 40 Å × 40 Å was set up surrounding the active site, with an exhaustiveness value of 8. The computing was done on the Linux-based system, and the pose holding the minimum binding energy value was chosen. Consistency was validated based on repeated cycles of docking, with RMSD values < 2.0 Å. Visualization and interaction analysis were done with Discovery Studio Visualizer 2021 for hydrogen bonds, hydrophobic interactions, and π-π stacking. Control docking with a known TGF-β inhibitor was performed to verify the protocol^29^.

### Statistical analysis

The statistical analysis was carried out using GraphPad Prism 10.0. Data were expressed as mean ± SEM. The normality of the data was assessed with the Shapiro-Wilk test. The one-way ANOVA test determined whether the difference among the groups was significant, followed by Tukey’s multiple comparison, which was used to check the difference in scores between different groups. *P*≤0.05 was considered statistically significant.

## Results

MSG, a widely used flavor enhancer in food products, has been involved in the pathogenesis of different organs^15,16^. Previously published data showed that MSG significantly increases the body weight of the rat via lipogenic activity, followed by dyslipidemia; however, quercetin acts as a lipolytic molecule to suppress the chances of MSG-induced obesity in rats by regulating lipid profile^17^. Moreover, no such impact on food habits was observed in the study. MSG acts by modifying the key cytokines and growth factors that include the effects of TGF-β, IL-17, IL-19, and VEGF on inflammation, the deposition of collagen, and angiogenesis, which lead to the characteristic tissue remodeling seen in fibrosis. It further emphasizes the maladaptive responses of the cells to hypoxic conditions that are worsened by treatment with MSG, with the enhanced expression of HIF-1α. At the same time, pro-fibrotic signals due to the unbalanced signaling and decreased PEDF and endostatin drive the phenotype toward fibrosis. The present research focuses on the natural flavonoid quercetin, characterized by its antioxidant and anti-inflammatory properties, which could be one potential modulator that can counteract pathophysiological mechanisms caused by MSG. Through the ability to restore MSG-induced altered signaling pathways and cytokine levels, quercetin reduces inflammation and fibrosis while maintaining the physiological homeostasis of the lung. This research explains the involvement of key cytokines and growth factors in MSG-induced lung fibrosis as well as the protective modulation by quercetin, which could mark great therapeutic potential in suppressing fibrotic responses.

### Role of key cytokines, growth factors, and Quercetin modulation on dysregulated signaling pathways in MSG-induced lung fibrosis

**Fig. 2:**
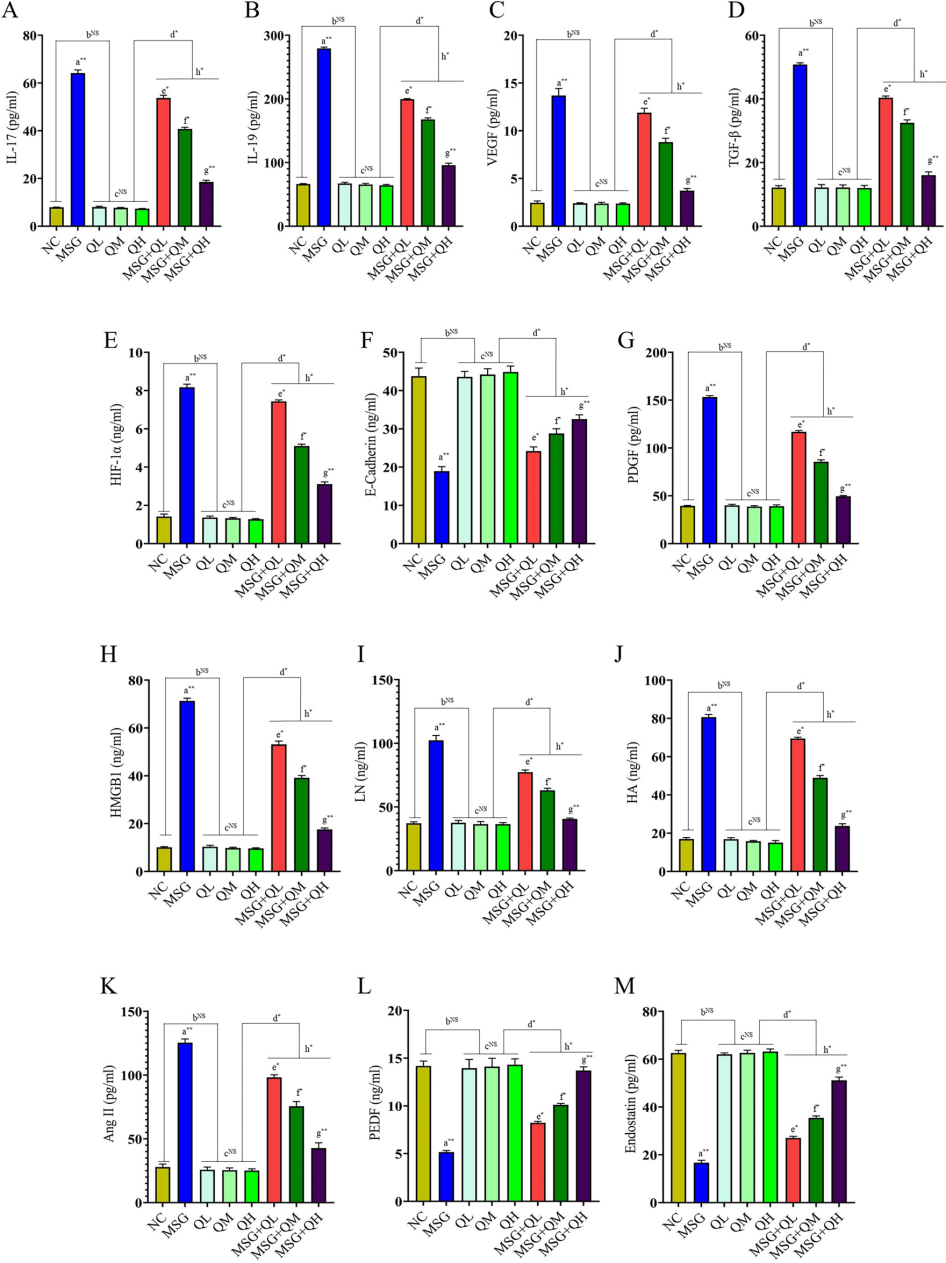
MSG-induced lung fibrosis: role of key cytokines, growth factors, and quercetin in modulating dysregulated signaling pathways (**A**-**G**). Quercetin regulates the equilibrium of pro and anti-fibrotic factors in MSG-fed rats (**H**-**M**). Data were expressed as Mean ± SEM. Significance level based on one-way ANOVA, *P* ≤ 0.05. Significance level based on Tukey’s post hoc test (*n* = 5): NC vs. MSG: a, NC vs. QL/QM/QH: b, QL vs. QM vs. QH: c, QL/QM/QH vs. MSG + QL/MSG + QM/MSG + QH: d, MSG vs. MSG + QL: e, MSG vs. MSG + QM: f, MSG vs. MSG + QH: g, MSG + QL vs. MSG + QM vs. MSG + QH: h [*P ≤ 0.05, **P ≤ 0.01, ***P ≤ 0.001, NS: not significant].

MSG provokes fibrosis in rats via a complex mechanism of different key signaling molecules. An increase (*P*≤0.01) in the level of IL-17 and IL-19 (Fig. 2A, B) in the MSG-fed group were observed in the study, which led to activate inflammation and tissue remodeling, followed by the progression of fibrosis^6,30^. In addition, an increase in VEGF (Fig. 2C) demonstrates increased angiogenesis^15^typically accompanied by fibrosis, which helps nourish and oxygenate the injured tissue. TGF-β is the major inducer of fibroblast activation and extracellular matrix deposition, which is important in the pathogenesis of pulmonary fibrosis^31^. MSG increases (*P*≤0.01) the level of TGF-β in lung tissue as compared to the NC group to promote fibrotic changes in the lung (Fig. 2D). Apart from these cytokines, increased (*P*≤0.01) levels of HIF-1α (Fig. 2E) in MSG-fed rats indicate that cells undergo adaptive changes towards cellular survival in low oxygen concentrations, which can therefore enhance fibrotic processes by regulating glycolysis and maintaining the survival of inflammatory cells under hypoxic conditions^32,33^.

Administration of MSG decreases (*P*≤0.01) serum levels of E-cadherin; it plays a significant role in maintaining epithelial integrity and preventing fibrosis^34^. There is a reversal of these changes, about decreased levels of E-Cadherin (Fig. 2F) and an increase in the activation of fibroblasts and collagen deposition that results in lung fibrotic changes, which were well corroborated with increased collagen and α-SMA levels in lung tissue, respectively. However, supplementation with the well-known antioxidative and anti-inflammatory flavonoid quercetin significantly recovered reduced (MSG vs. MSG + QL/QM: *P*≤0.05, MSG vs. MSG + QH: *P*≤0.01) serum E-Cadherin levels. The protective mechanism of quercetin also involves the modulation of signaling pathways that are associated with epithelial-to-mesenchymal transition (EMT) and decreases activation of fibroblasts, thereby re-establishing a normal cellular microenvironment. Furthermore, an increased (*P*≤0.01) level of PDGF (Fig. 2G) in the MSG-fed group also contributes to fibrosis by attracting fibroblasts toward an injury site and subsequently stimulating the proliferation of fibroblasts. These markers of alteration in the extracellular matrix are also indicators of cellular stress and damage, such as HMGB1 (Fig. 2H), LN (Fig. 2I), and HA (Fig. 2J) in the MSG group. Ang II increase (Fig. 2K) is unique because it not only increases blood pressure but also stimulates fibrogenesis, thus linking vascular alterations with the fibrotic phenotype^35^which also increases (*P*≤0.01) in MSG-fed rats as compared to the NC group. On the other hand, it has been demonstrated that rats fed on MSG express reduced (MSG vs. MSG + QL/QM: *P*≤0.05, MSG vs. MSG + QH: *P*≤0.01) levels of anti-fibrotic and anti-angiogenic factors PEDF (Fig. 2L) and endostatin (Fig. 2M). The factor fails the tissue repair processes and thus helps prevent tissue injury. Therefore, the imbalance in pro-fibrotic and anti-fibrotic signals is the direct driving force behind the phenomenon of lung fibrosis in that particular animal model. It is also fascinating that quercetin, a natural flavonoid with antioxidant and anti-inflammatory properties, apparently affords some protection against MSG-induced lung fibrosis.

Quercetin’s beneficial effect probably acts by modulating the very same pro-inflammatory cytokines and growth factors elevated by MSG. In higher doses (MSG vs. MSG + QL/QM: *P*≤0.05, MSG vs. MSG + QH: *P*≤0.01), quercetin has more pronounced attenuation of the inflammatory response and fibrotic changes, confirmed by restoring normal serum levels of the cytokines and the growth factors involved in fibrogenesis. Interestingly, quercetin alone did not show marked changes compared to the normal control (NC vs. QL/QM/QH: NS), whereas it can antagonize the effects of MSG (MSG vs. MSG + QL/QM: *P*≤0.05, MSG vs. MSG + QH: *P*≤0.01); which indicates that it would not independently influence lung health without injury and regulating the signaling pathways in lung fibrosis through which such pathologies may be mitigated by potential therapeutic interventions such as quercetin.

### Quercetin as a signaling modulator in MSG-induced lung fibrosis and extracellular matrix (ECM) remodeling

**Fig. 3:**
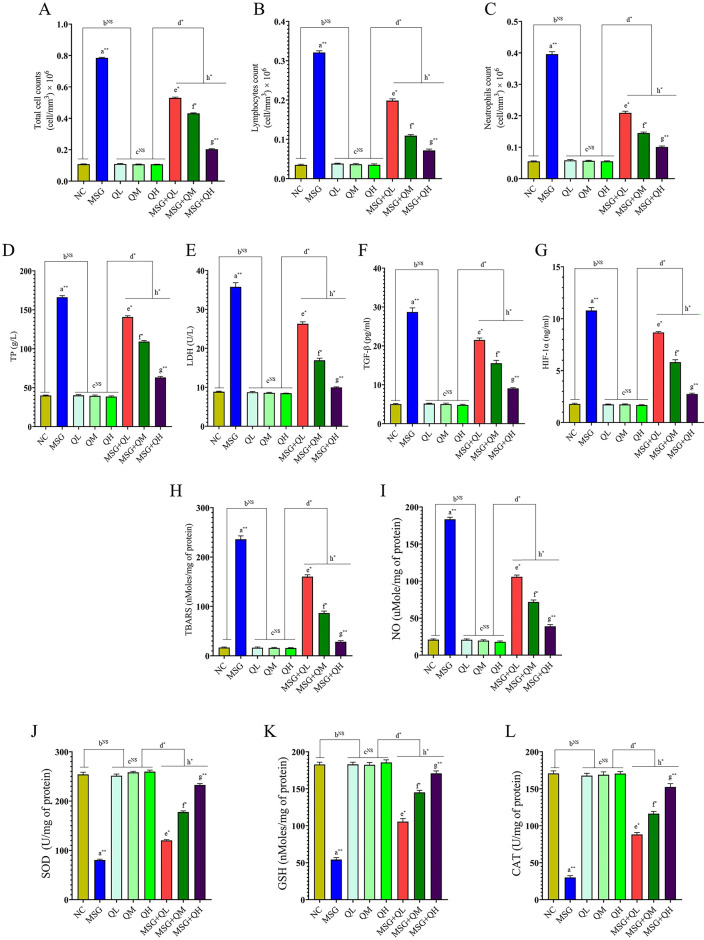
MSG-induced pulmonary fibrosis and ECM remodeling were modulated by quercetin (**A**-**M**). Cross-talk between oxidative stress and pulmonary fibrosis: possible protection through quercetin against MSG-incited changes in the lung (**H**-**L**). Data were expressed as Mean ± SEM. Significance level based on one-way ANOVA, *P* ≤ 0.05. Significance level based on Tukey’s post hoc test (*n* = 5): NC vs. MSG: a, NC vs. QL/QM/QH: b, QL vs. QM vs. QH: c, QL/QM/QH vs. MSG + QL/MSG + QM/MSG + QH: d, MSG vs. MSG + QL: e, MSG vs. MSG + QM: f, MSG vs. MSG + QH: g, MSG + QL vs. MSG + QM vs. MSG + QH: h [*P ≤ 0.05, **P ≤ 0.01, ***P ≤ 0.001, NS: not significant].

MSG caused lung fibrosis in male rats primarily due to an elevation (*P*≤0.01) in total, lymphocytes, and neutrophil cell count (Fig. 3A-C). Quercetin was protective against lung damage, with an increasing dose, such that higher doses of quercetin showed a greater protective effect compared with the group treated with MSG (MSG vs. MSG + QL/QM: *P*≤0.05, MSG vs. MSG + QH: *P*≤0.01). Notably, only quercetin alone did not show significant changes as compared to the normal control group (NC vs. QL/QM/QH: NS); hence, though quercetin is active against MSG-induced damage in the lungs, it has a basal protective action under normal physiological conditions. The signaling pathways in cells are involved in these mechanisms of action. An ability to activate inflammatory signaling cascades in cells could lead to enhanced pro-inflammatory cytokines and infiltration into the lung tissue, causing the proliferation of fibroblasts and resultant depositions of ECM typical of lung fibrosis^36^. Possibly quercetin modulates the cellular signaling pathways to inhibit the activation of the inflammatory mediators by enhancing antioxidant responses, which would help reduce oxidative stress and inflammation. Therefore, quercetin seems to neutralize the pathological effects of MSG through complex interactions with cellular signaling involved in inflammation and fibrosis.

### Role of MSG in lung injury and protective potential of quercetin: Deciphering fibrotic response

The present work shows that MSG causes lung fibrosis , where it increases (*P*≤0.01) major inflammatory and fibrotic markers in the bronchoalveolar lavage fluid of male rats. Elevated total protein and lactate dehydrogenase (Fig. 3D, E), which indicated cellular damage and inflammation, and increased levels of TGF-β and HIF-1α that suggested the activation of fibrotic pathways and cellular responses to hypoxia, provided evidence that TGF-β is highly implicated in enhancing the activation of fibroblasts and collagen deposition and, therefore, tissue fibrosis (Fig. 3F). In addition, HIF-1α (Fig. 3G) play an important role in hypoxic adaptation at the cellular level while directly fibrotic processes through stimulation of angiogenic and extracellular matrix-related pathways.

In contrast, quercetin offers protective effects on the integrity of the lungs in this model. Indeed, important benefits are found at higher doses. Quercetin significantly reduces lung fibrosis and inflammation in MSG-fed rats. All graded increasing doses of quercetin resulted in positive effects, but the maximum protection was observed at the highest dosage (MSG vs. MSG + QL/QM: *P*≤0.05, MSG vs. MSG + QH: *P*≤0.01) compared with the MSG-treated group, indicating a dose-dependent response for its protective mechanism. However, noteworthy in this regard is that within the quercetin subgroup alone, no change reaching statistical significance (NC vs. QL/QM/QH: NS) was found when compared with the corresponding NC group, which implies that quercetin has no adverse effects or alterations in lung function, at least under non-fibrotic, healthy conditions. The results thus indicate how MSG contributes towards inducing fibrosis in the lung by promoting inflammatory signaling pathways, but demonstrate the possibility of a protective mechanism with the use of quercetin counteracting these pathological processes.

### Oxidative stress plays a silent role in the development of MSG-induced lung fibrosis: Dose-dependent protective role of Quercetin

MSG is known to induce oxidative stress^17^which ultimately increases the chances of fibrosis of the lung, mainly through the induction of the accumulation process of the ECM components due to the EMT^37^. This response may be induced by elevated (*P*≤0.01) levels of lipid peroxidation products that include TBARS (Fig. 3H) and NO (Fig. 3I). Concurrently, there is a significant reduction (*P*≤0.01) of major antioxidants SOD, GSH, and CAT (Fig. 3J-L) in the lung tissues of male rats. These biochemical changes demonstrated disturbances in free radicals/antioxidants levels to be the central mechanism in the pathogenesis of oxidative damage at the cellular level caused by MSG in the present study, which was well corroborated with previous reports^17,38^; this can also lead to serious lung injury, followed by fibrotic responses.

However, the effect of quercetin on MSG-induced oxidative damage in the lung was a significant protective effect; therefore, it can be stated that quercetin acts as an antioxidant molecule against MSG-induced oxidative damage in line with earlier studies^17,18^. The highest dose of quercetin provided the most significant (MSG vs. MSG + QL/QM: *P*≤0.05, MSG vs. MSG + QH: *P*≤0.01) attenuation of the adverse effects compared to the treated group. On the contrary, quercetin alone did not have alterations significantly (NC vs. QL/QM/QH: NS) different from the NC group, which suggests that although it is very potent in mitigating oxidative damage caused by MSG , it does not have any effect on the normal baseline conditions of lung tissue.

### The anti-fibrotic nature of Quercetin in MSG-induced lung fibrosis by targeting the TGF-β pathway

**Fig. 4:**
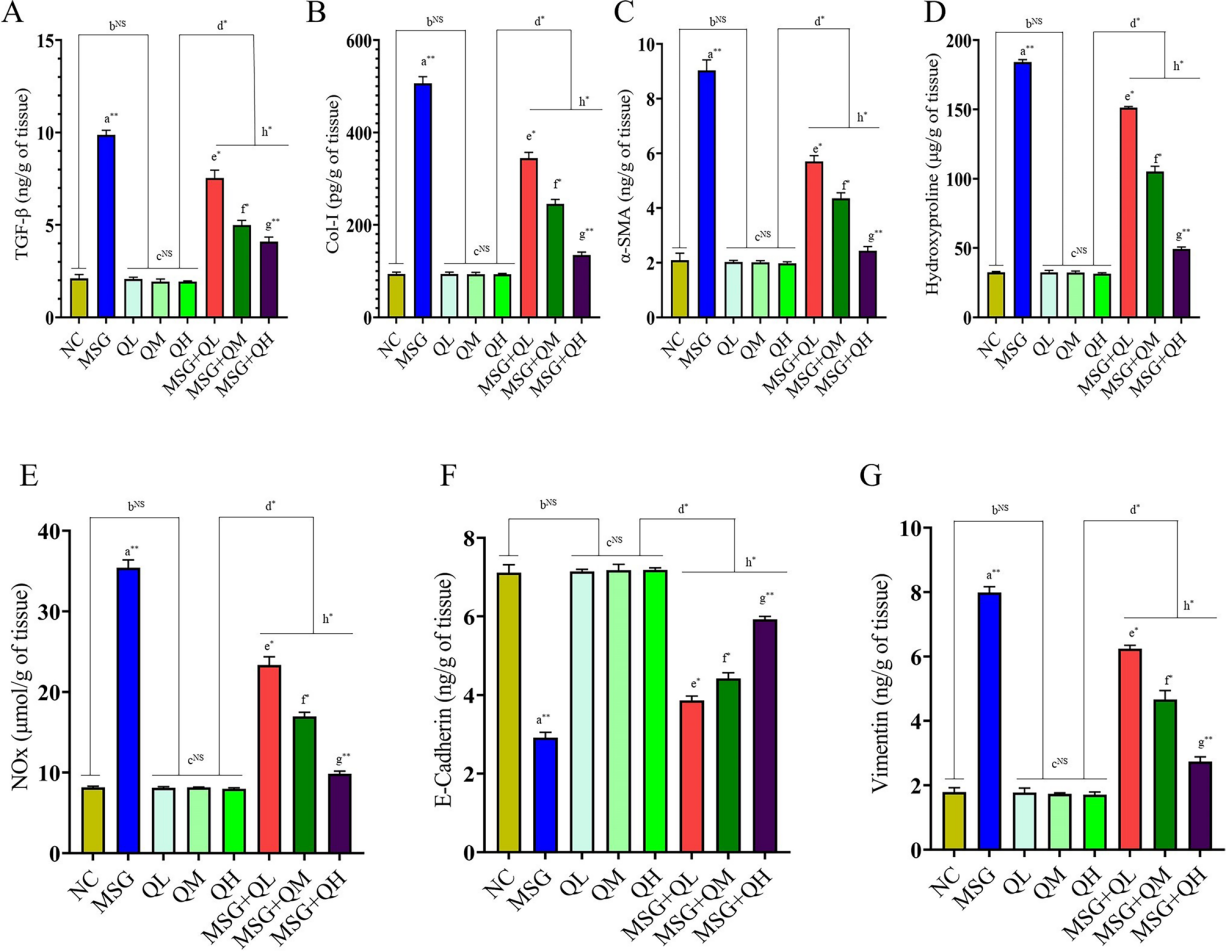
Quercetin as an anti-fibrotic molecule blunted the occurrence of MSG-induced lung fibrosis via suppressing TGF-β (**A**-**G**). Data were expressed as Mean ± SEM. Significance level based on one-way ANOVA, *P* ≤ 0.05. Significance level based on Tukey’s post hoc test (*n* = 5): NC vs. MSG: a, NC vs. QL/QM/QH: b, QL vs. QM vs. QH: c, QL/QM/QH vs. MSG + QL/MSG + QM/MSG + QH: d, MSG vs. MSG + QL: e, MSG vs. MSG + QM: f, MSG vs. MSG + QH: g, MSG + QL vs. MSG + QM vs. MSG + QH: h [*P ≤ 0.05, **P ≤ 0.01, ***P ≤ 0.001, NS: not significant].

The study established the effects of MSG on lung fibrosis and different biomarkers in the connection of cellular signaling pathway. It was demonstrated that the MSG causes a significant elevation (*P*≤0.01) in the level of TGF-β (Fig. 4A), which encourages the activation of fibroblasts and, subsequently, collagen deposition. Moreover, higher (*P*≤0.01) levels of Col-I (Fig. 4B) as a major component of the extracellular matrix and α-SMA (Fig. 4C) increased fibrosis and myofibroblast differentiation and contributed to tissue remodeling in MSG-fed rats. In addition, MSG elevates (*P*≤0.01) both hydroxyproline (Fig. 4D), and the total NOx (Fig. 4E) levels; this suggests increased collagen turnover coupled with increased production of reactive nitrogen species that could further promote oxidative stress and inflammation.

On the other hand, the study found a decreased (*P*≤0.01) level of E-Cadherin (Fig. 4F), an important epithelial cell adhesion molecule in MSG-fed rats, suggesting there is an impairment in epithelial integrity and may even support the process of EMT. E-Cadherin is a major cell adhesion marker; its decreased expression can lead to favorable conditions for the development of fibrosis ^42; ^ Therefore, MSG affects signaling pathways causing fibrosis through several mechanisms, including its effects on cadherins as well as on components of the ECM. Furthermore, administration of MSG caused a significant increase (*P*≤0.01) in the levels of vimentin in lung tissues (Fig. 4G), therefore suggesting the induction of the fibrosis pathway. Increased vimentin, an EMT and fibrosis marker, was significantly attenuated by quercetin administration, restoring the levels of vimentin to normalcy.

**Fig. 5:**
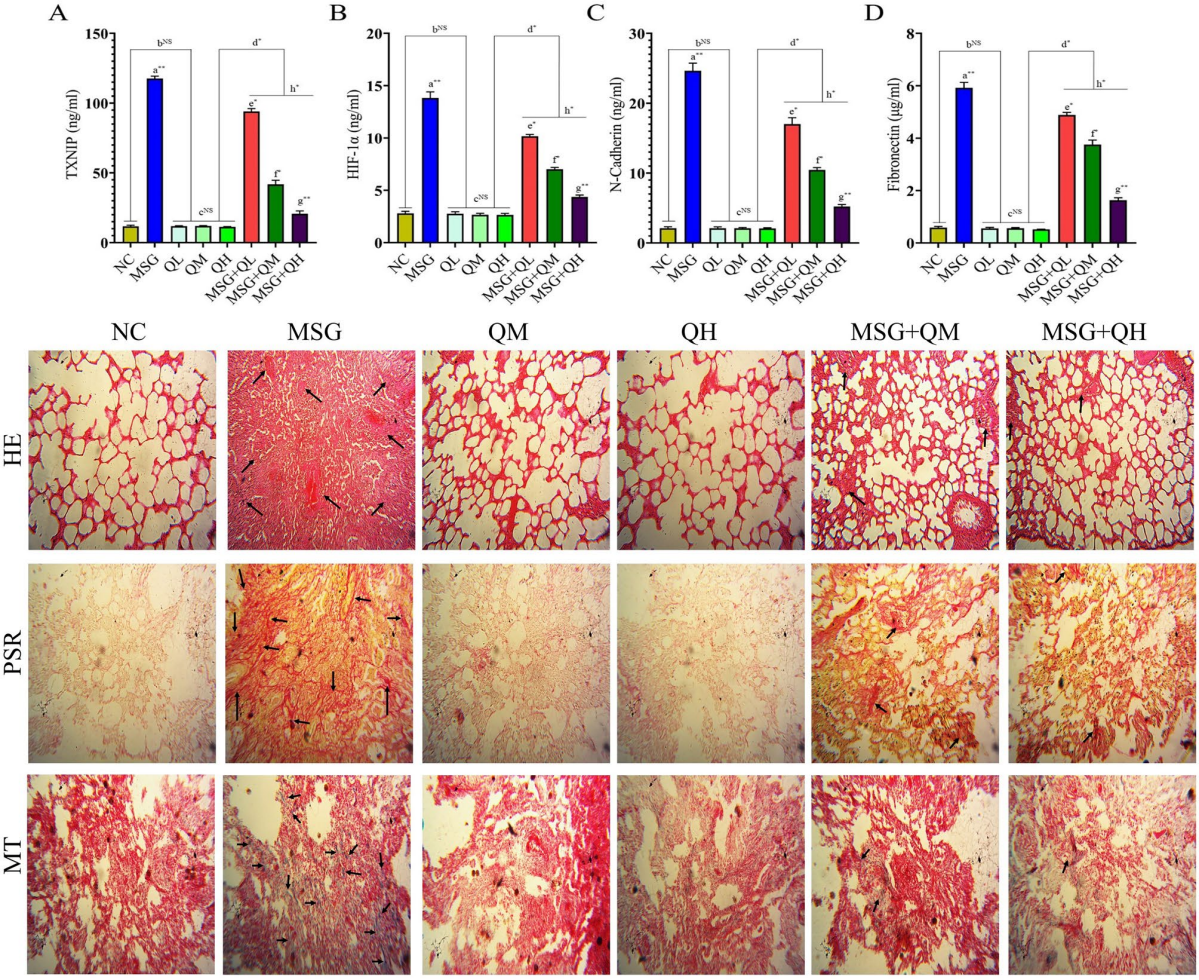
TXNIP and inflammatory pathways: Role of MSG and quercetin in the development of pulmonary fibrosis through balance in redox and myofibroblast differentiation (**A**-**D**). Data were expressed as Mean ± SEM. Significance level based on one-way ANOVA, *P* ≤ 0.05. Significance level based on Tukey’s post hoc test (*n* = 5): NC vs. MSG: a, NC vs. QL/QM/QH: b, QL vs. QM vs. QH: c, QL/QM/QH vs. MSG + QL/MSG + QM/MSG + QH: d, MSG vs. MSG + QL: e, MSG vs. MSG + QM: f, MSG vs. MSG + QH: g, MSG + QL vs. MSG + QM vs. MSG + QH: h [*P ≤ 0.05, **P ≤ 0.01, ***P ≤ 0.001, NS: not significant]. The cytoarchitectonic view of lung tissues was also depicted in this figure, which shows the restorative impact of quercetin in the MSG-induced altered cytomorphology by HE (HE), Picrosirius red (PSR), and Masson’s trichrome (MT) staining. Histological examination of lung tissue in NC, MSG, and two graded concentrations of quercetin with or without MSG showed the variants in terms of structure integrity and fibrotic responses. Normal alveolar structures with minimal inflammatory infiltration were seen in the NC group by hematoxylin and eosin staining (HE). On the other hand, the MSG group presented intense pulmonary fibrosis, whose histological appearance was disturbed due to higher collagen deposition and inflammatory cell infiltration in all of the used staining methods. Interstitial edema and inflammation were mainly emphasized under HE staining (HE), whereas Picrosirius red staining (PSR) indicated the existence of thick fibers that represent fibrotic remodeling. Areas stained with Masson’s trichrome revealed an increase compared with controls (MT). The treatment at two concentrations of quercetin has not shown any alterations of the lung histology compared with the NC group, indicating that this compound has limited or no significant influence on the lung independently. However, quercetin + MSG offered protective and significantly reduced the level of fibrotic changes with all the stainings ; thus, there is evidence that although quercetin itself does not produce marked remodeling of lung tissue, it effectively rescues from the pathological effects of MSG and saves the structure and attenuates inflammatory responses.

Additionally, TXNIP link to cellular redox signaling and is implicated in inflammatory and fibrotic processes^39^which was higher (*P*≤0.01) in the MSG-fed group (Fig. 5A). Moreover, increased (*P*≤0.01) HIF-1α (Fig. 5B) related to the cellular response to promote fibrotic pathways with hypoxia^31^accompanied by a concurrent increase (*P*≤0.01) of N-Cadherin (Fig. 5C)^40^. The results suggest increased cell adhesion and contribute to myofibroblast differentiation, which might further increase the chances of lung fibrosis in MSG-fed rats. Notably, the elevation of TGF-β play a pivotal role in the fibrotic process due to enhanced deposition of extracellular matrix and myofibroblast activation^41,42^. Altogether, the factors of the pathogenesis of lung fibrosis in the induction of altered cellular signaling are enhanced by MSG treatment.

Furthermore, administration of MSG to rats resulted in a significant (*P*≤0.01) elevation in the levels of fibronectin (Fig. 5D) in the lung; this further suggests that there would be a predisposition toward fibrotic changes due to the activation of signaling pathways like TGF-β and resultant stimulation of extracellular matrix deposition^43^. The enhancement of the levels of one of the major mediating proteins during fibrosis, such as fibronectin, leads to increased development of pulmonary fibrosis. Quercetin treatment suppressed (MSG vs. MSG + QL/QM: *P*≤0.05, MSG vs. MSG + QH: *P*≤0.01) the higher levels of fibronectin associated with MSG-fed rats through its antioxidant activity and inhibition of the TGF-β signaling pathway, thus suppressing oxidative and inflammatory stress factors that contribute to fibrotic progression. The protective mechanism appeared to be linked with its antioxidant properties, thereby moderating oxidative stress and inflammation, which would inhibit the activation of the fibrogenic signaling cascades induced by MSG. Quercetin, therefore, would not only normalize the content of pro-fibrogenic markers but also maintain the lung architecture, suggesting a potential therapeutic role in the prevention of pulmonary fibrosis secondary to exposure to MSG. Therefore, it can be stated that quercetin treatment significantly improved lung biomarkers altered by MSG. All three graduated doses of quercetin significantly alleviated the effects of MSG-induced damage, exhibiting a stronger protective effect with the higher dosages (MSG vs. MSG + QL/QM: *P*≤0.05, MSG vs. MSG + QH: *P*≤0.01). Thus, quercetin may act as an anti-fibrotic through the modification of the pathways described above and via the suppression of pro-fibrotic markers. However, quercetin alone did not produce significant alterations in the biomarkers when compared with the normal control; thus, its protective effects are only, as observed here, related to MSG-induced lung injury.

Hence, it suggests that MSG causes lung fibrosis through pro-fibrotic TGF-β and related markers signaling pathways by increasing collagen deposition and the activation of myofibroblasts. Quercetin exhibited a cytoprotective effects against MSG-induced lung damage, possibly due to its modulation of cell signaling pathways that are involved in fibrosis, inflammation, and oxidative stress. Therefore, these findings draw attention to the complex interplay of cell signaling pathways in the development of lung fibrosis and present quercetin as a potential therapeutic agent for the attenuation of pathological changes induced by MSG.

### Modulatory role of Quercetin in MSG-induced histological alteration in the promotion of fibrotic response

Histological analyses of lung tissues in the performed study after various treatments, including NC, MSG, and two increasing graded doses of quercetin (QM and QH as per the statistical significance level and previous data) with and without MSG, showed differences regarding structural integrity and fibrotic responses. NC showed relatively normal histological architecture in HE staining (Fig. 5HE), indicating normal lung tissue with normal alveolar structure and minimum inflammatory infiltration. However, the MSG group displayed characteristics of pulmonary fibrosis; it demonstrated high collagen accumulation and inflammatory cell infiltration in all histological stains. Most of the changes in this study were noted in HE staining, with marked interstitial edema and inflammatory cell infiltration. Thick fibers that represent fibrotic remodeling were obtained with Picrosirius red (Fig. 5PSR), whereas Masson’s trichrome (Fig. 5MT)-stained areas were nearly doubled compared to the controls.

When quercetin was evaluated at two different concentrations, it was reported that the histology of the lungs was not affected as compared to the NC group, which could indicate that Quercetin cannot independently affect the nature of the lungs. Quercetin, however, combined with MSG, showed a protective effect against damage induced by MSG in histological examination; degrees of fibrotic changes were significantly reduced with higher doses of quercetin as reflected in all the stainings . Thus, it could help provide evidence that though Quercetin itself did not cause significant tissue remodeling, it effectively countered the pathological effects of MSG, thus restoring structural integrity and reducing the inflammatory responses.

The histological changes can be directly correlated with the underlying mechanisms of cytokine and growth factor signaling. MSG upregulated several mediators that contribute to inflammation, hypoxia, and fibroblast activation: IL-17, IL-19, TGF-β, and VEGF. Such mediators promote extracellular matrix deposition and an increase in angiogenesis^44–46^; therefore, such factors produce the vicious cycle of fibrosis. In particular, TGF-β signals were essential in promoting the proliferation of fibroblasts and collagen synthesis, while the HIF-1α signals cellular adaptation in low-oxygen conditions that further support the development of fibrosis^47^. The MSG group shows an increased level of TXNIP, while the lower levels of E-Cadherin translate to cellular stress along with the disruption of epithelial integrity, increasing the fibrotic phenotype.

On the other hand, quercetin presented a modulatory function in terms of the downregulation of pro-inflammatory cytokines and promotion of the balance toward antifibrotic signaling. It was also acting through the improvement of antioxidant defenses; the markers of oxidative stress were being reduced, and over-activated pathways due to MSG were being reduced. More particularly, at higher doses, Quercetin normalized serum levels of TGF-β, IL-17, and IL-19, thus improving the fibrotic environment by reducing pro-fibrotic signaling and homeostasis in the extracellular matrix. Therefore, in a nutshell, the complex interaction of histological changes and cytokine signaling clarifies how MSG leads to fibrosis in the lungs by disrupting the network of the key pathways while underlining Quercetin’s potential as an effective drug in fighting such pathologies by finding the pro-anti-fibrotic balance that thus protects lung architecture.

### Quercetin hinders Ang II/AT1 to ameliorate the MSG-induced fibrotic response in the lung by targeting the TGF-β/Smad pathway

**Fig. 6:**
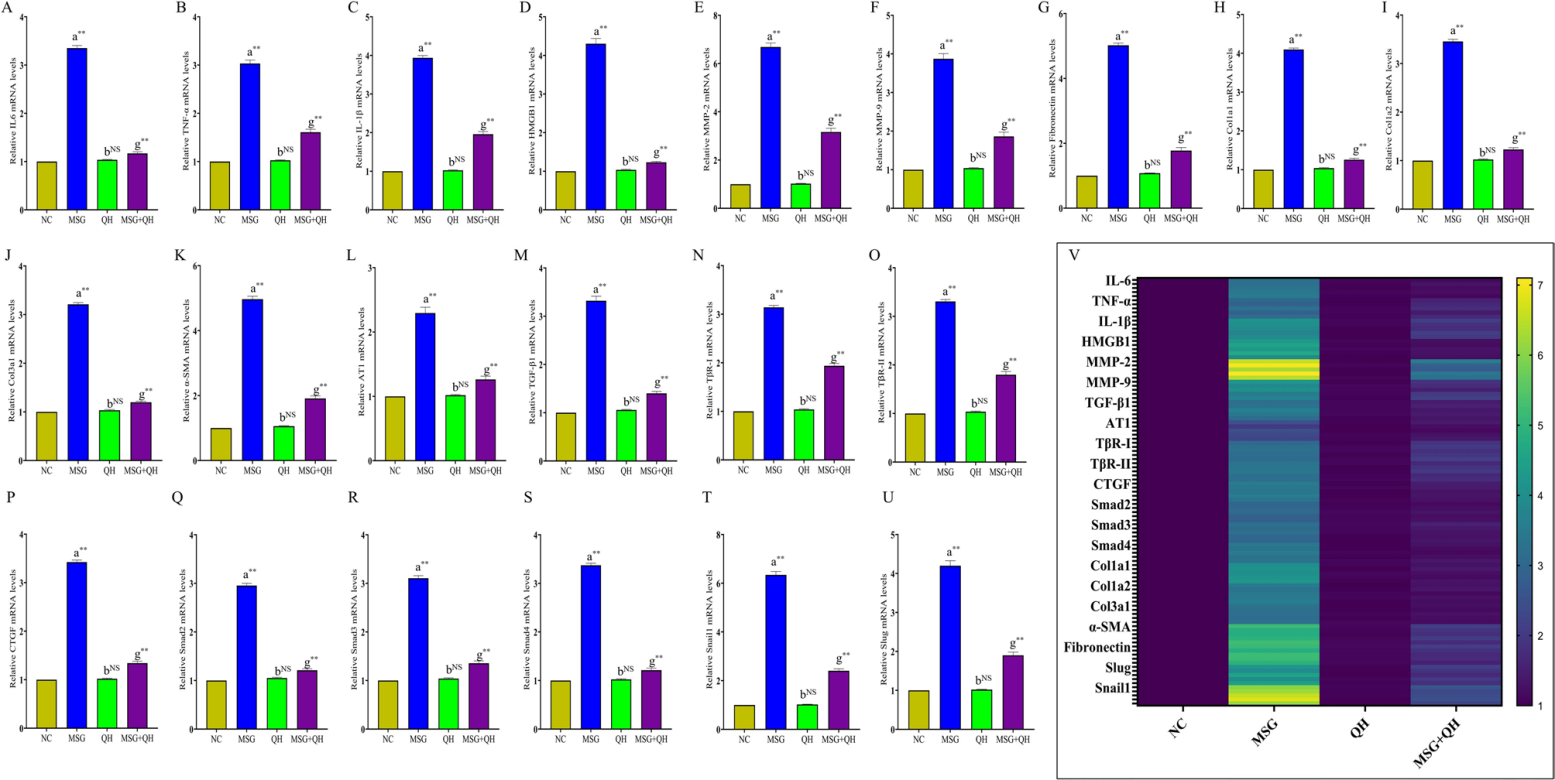
Role of quercetin in MSG-induced changes in pro-inflammatory and pro-fibrotic gene expression (gene expression:** A**-**U**; heatmap:** V**) by regulating AT1 and TGF-β/Smad pathway. Data were expressed as Mean ± SEM. Significance level based on one-way ANOVA, *P* ≤ 0.05. Significance level based on Tukey’s post hoc test (*n* = 5): NC vs. MSG: a, NC vs. QL/QM/QH: b, MSG vs. MSG + QH: g [**P ≤ 0.01, NS: not significant].

In this part of the experimental setup, the present work only selects the NC, MSG, QH, and MSG + QH groups as per the previous data and statistical significance level of the impact on different parameters. The results show that MSG promotes a pro-inflammatory response with elevated (*P*≤0.01) levels of IL-6, TNF-α, and IL-1β (Fig. 6A-C). These cytokines are more inflammatory and have been demonstrated to increase fibrosis through signaling molecules like HMGB1 (Fig. 6D, *P*≤0.01), which enhance cellular responses to injury and fibrotic stimuli^48^. In addition, MMP-2 and MMP-9 (Fig. 6E, F) upregulation (*P*≤0.01), along with part of the extracellular matrix: fibronectin (Fig. 6G) and collagen, e.g., Col1a1, Col1a2, and Col3a1 (Fig. 6H-J), moreover, MSG promotes vascular remodeling and fibrosis by mechanisms of stimulation of collagen deposition and the remodeling of the matrix, and induction of α-SMA (Fig. 6K) demonstrates differentiation of fibroblasts to myofibroblasts, which is an essential process in fibrosis development.

Concurrently, MSG has been implicated as being important in the upregulation of key markers and signaling pathways involved in fibrosis-related processes that contribute to the process of lung fibrosis. This occurred through the activation of the angiotensin II type 1 receptor (Fig. 6L), which, when stimulated with MSG, enhanced the expression of connective tissue growth factor, an important mediator in fibrotic processes. The upregulation of TGF-β1 (Fig. 6M), as a potent fibrogenic promoter to initiate signaling via TβR-I (Fig. 6N) and TβR-II (Fig. 6O), and the upregulation of CTGF (Fig. 6P) (*P*≤0.01) is further stressed. This cascade activates Smad 2 (Fig. 6Q), Smad 3 (Fig. 6R), and Smad 4 (Fig. 6S), which become translocated to the nucleus for the regulation of the expression of several pro-fibrotic genes, including Snail and Slug (Fig. 6T, U). The heatmap shows the overall gene expression changes by MSG with or without quercetin (Fig. 6V).

On the other hand, quercetin plays a multifunctional protective role against MSG-induced lung fibrosis by targeting the Ang II/AT1 signaling axis. It thereby inhibits Ang II/AT1, which was well corroborated with earlier work^49^ and results in the significant reduction of the pro-fibrotic signaling initiated by MSG. It acts as an inhibitor affecting the downstream activation of the TGF-β/Smad signaling, thus suppressing the upregulation of the factors that involve CTGF, TGF-β1, and α-SMA in fibrosis^50,51^. Furthermore, the anti-inflammatory action of quercetin reduces (*P*≤0.01) the production of inflammatory cytokines, such as IL-6, IL-1β, TNF-α, and HMGB1, that otherwise exacerbate the fibrotic process. More importantly, the inhibition by quercetin of crucial ECM components and MMPs is crucial in maintaining the lung architecture, possibly a reason for the development of MSG-induced pulmonary fibrosis as a potential therapeutic agent. Quercetin was, therefore, demonstrated to modulate profibrotic and anti-fibrotic pathways as it reverted the fibrotic changes and appeared to protect the lung tissue against MSG-induced damage.

### Docking analysis: MSG-mediated activation of TβR-II and quercetin-mediated Inhibition of MSG-activated TβR-II

**Fig. 7:**
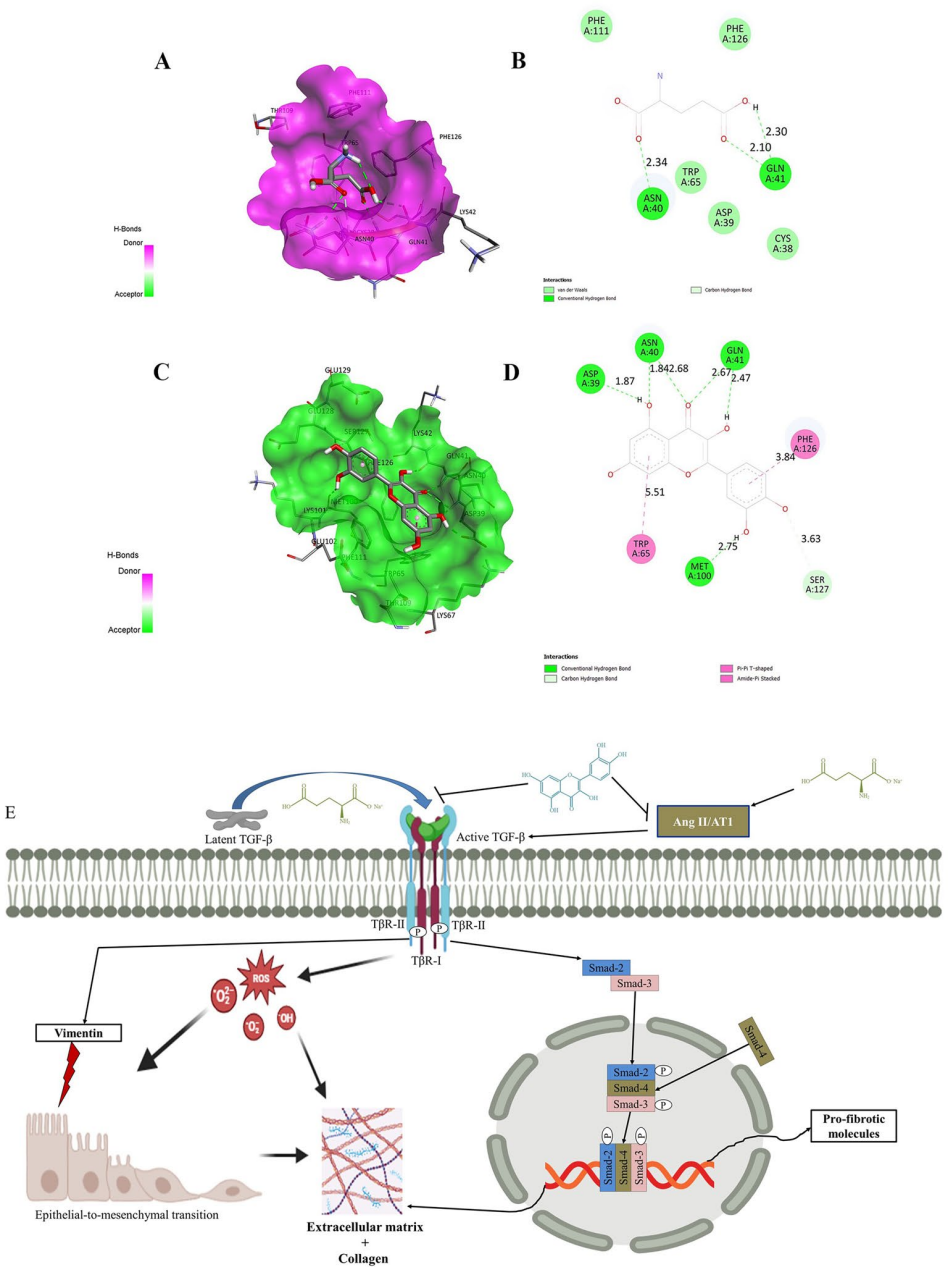
Molecular docking analysis of MSG and quercetin with TβR-II (AutoDock Vina and Discovery Studio were used to make the figure). (**A**) The 3D interaction of MSG with the TβR-II, as shown by Discovery Studio Visualizer, is described in terms of surface and major interacting residues. (**B**) A 2D diagram for classic hydrogen and carbon-hydrogen bonds between MSG and other residues of TGF-β RII such as Asn40, Gln41, and Trp65. (**C**) Representation of the 3D illustration of the binding interaction of quercetin with TβR-II. In this illustration, the receptor is shown to be surficial towards the involvement of residues important in the interaction. (**D**) 2D map of quercetin interaction, with hydrogen bonds, Pi-Pi T-shaped and Amide-Pi stacking interaction modes towards the key residues involved: Asn40, Gln41, Trp65, Phe126. The interaction distances are given in Å. (**E**) The mechanistic overview of MSG on lung to cause fibrotic responses by activating TGF-β/Smad and the role of quercetin in such anomalous situation (BioRender was used to make the figure: https://biorender.com).

It is found that there are significant interactions in the molecular docking study between MSG and TβR-II, which implies that MSG can control the receptor by activating downstream pathways like SMAD. The interaction was calculated to have a binding affinity of − 4.2 kcal/mol. Also, the RMSD value came out to be 1.292 Å, indicating an efficient and stable pose of the binding. Significant hydrogen bonds were detected between MSG and the residues Asn40, Gln41, and Trp65. It is interesting to note that MSG forms hydrogen bonds with Asn40 at a distance of 2.354 Å, which may stabilize the ligand inside the active site of the receptor. In addition to the two hydrogen bonds with Gln41, distances of 2.106 Å and 2.304 Å were present, which reveal that Gln41 plays a vital role in the anchoring of MSG and the structural changes that must take place to activate the receptor. A weaker carbon-hydrogen bond at 3.085 Å with Trp65 further stabilizes but is compensated by another weak hydrogen bond with Cys38 at 3.431 Å. Angles of hydrogen bonds were near 180°, in the examples 164.93° and 162.61°, which would imply very stable and directional interactions favorable for receptor activation (Fig. 7A, B). The 3D visualization shows that the binding pocket of the receptor in which MSG fits so well is surrounded by residues Asn40, Gln41, and Trp65, along with hydrophobic aromatic residues Phe111 and Phe126; these further stabilize the complex through van der Waals interactions. This tight binding alignment suggests that MSG could induce conformational shifts within the receptor, which is the main step in receptor dimerization and SMAD protein phosphorylation. The moderate binding affinity of − 4.2 kcal/mol supports the hypothesis that the interaction is biologically relevant, and a low value of the RMSD, about 1.292 Å, confirms the precision of the docking results. Interaction with residues such as Asn40 and Gln41 in regions of critical functional domains of the receptor could suggest a role for MSG in the activation of the receptors. These residues play a strategic role because they are located at a point where they influence the dynamics of receptor conformation, which subsequently leads to downstream consequences concerning the pathway of SMAD. Other hydrophobic interactions with Phe111 and Phe126 provide further stability to the interaction, such that when the complex is formed, it is less likely to dissociate. From these results, it would appear that MSG acts as a natural ligand, where activation of the TGF-β receptor (TβR) results in downstream signaling.

The molecular docking analysis of quercetin with TβR-II indicates a strong inhibitory interaction with a binding affinity of − 6.2 kcal/mol and an RMSD value of 7.026 Å, supporting the indication of significant receptor modulation. A qualitative, detailed interaction analysis reveals multiple hydrogen bonds, carbon-hydrogen bonds, and several Pi-stacking interactions that must exist for quercetin to bind with TβR-II. Quercetin, being hydrophilic, stabilizes the flavonoid in the receptor’s binding pocket by forming strong hydrogen bonds with important residues: Asn40 at 1.84 Å and Gln41 at 2.47 Å and 2.67 Å. A hydrogen bond with Asp39 at 1.87 Å and a carbon-hydrogen bond with Met100 at 2.75 Å add to the interaction (Fig. 7C, D). In the 3D visualization, it can be seen that quercetin extensively lies in the receptor hydrophobic pocket (Fig. 7C), where some residues encircle it, such as Phe126, Trp65, and Ser127. Its binding potency can be improved through a Pi-Pi T-shaped interaction with Trp65 at a distance of 5.51 Å and a secondary amide-Pi stacking with Phe126 at a distance of 3.84Å, which induces inhibition of receptor activity activation. The Pi-stacking interactions enforce the binding of quercetin by producing a favorable environment through aromatic residue interactions, which prevent receptor activation. The docking data also indicate that quercetin effectively competes with MSG for the same or overlapping binding sites on the receptor, but its stronger affinity (− 6.2 kcal/mol) suggests it can override the receptor’s activation state induced by MSG. The increased RMSD value of 7.026 Å compared to the MSG-TβR-II complex indicates a significant conformational change in the receptor upon quercetin binding, possibly leading to inhibition of receptor dimerization and disruption of Smad signaling. The interaction pattern suggests that quercetin binding stabilizes the receptor in an inactive conformation by altering the binding pocket dynamics through multiple hydrogen bonds and Pi-stacking interactions. These structural changes hinder the receptor’s ability to propagate downstream signaling, thus potentially inhibiting the pathway that MSG would have activated.

## Discussion

MSG has increasingly been recognized not only as a widely used flavor enhancer in food products but also as a compound implicated in adverse health outcomes^16,52, ^ one of the most serious consequences being pulmonary fibrosis. Cellular mechanisms that take place in MSG-induced fibrotic pathology must be complex since they involve major shifts in cellular signaling pathways relating to inflammation, collagen deposition, and tissue remodeling. At the core of this process of fibrosis lie several major cytokines, such as TGF-β, interleukins (IL-17, IL-19), and VEGF, including HIF-1α^6,32,44,53^.

MSG mainly acts through its interference with the balance between pro-fibrotic and anti-fibrotic pathways^15,54^. The induction of high levels of IL-17 and IL-19 after exposure to inflammatory stimulants like MSG triggers intense inflammatory reactions, while VEGF causes neovascularization, hence supporting the tissues under inflammation^55,56^. Additionally, TGF-β is an important factor since it acts as a major inducer of activation and ECM deposition for fibroblasts, playing pivotal roles as a central mediator that affects the conversion of fibroblasts into myofibroblasts in the course of fibrotic progression^41,57^. Simultaneously, an elevated HIF-1α level suggests that MSG enhances hypoxic adaptations, which often activate the glycolytic pathway and, through cell survival stress, promote cell growth and enhance fibrotic responses because of the increased persistence of inflammatory cells^58^.

Moreover, other signaling molecules, PDGF and Ang II, also make significant contributions to the same processes by recruiting and activating fibroblasts, which significantly accelerate the production of collagen^59,60^. In addition, there was a significant reduction in the level of protective factors like PEDF and endostatin in rats treated with MSG, signifying a severe dysfunction in the cellular signaling that causes the fibrotic response^10,11^. With subsequent imbalance between pro-fibrotic and anti-fibrotic signals, along with increased markers of oxidative stress such as TBARS and NO, this pathway ultimately facilitates the development of lung fibrosis^1^.

On the other hand, it is well known that quercetin is one of the naturally occurring flavonoids possessing very potent antioxidant and anti-inflammatory properties^17,18^. It has the potential to reverse the injurious effects caused by MSG^17^. Modulating different major pro-inflammatory cytokines and restoring balance within the altered signaling pathways have been an important aspect of the therapeutic promise offered by quercetin. The present study demonstrated that quercetin (mainly 100 mg/kg body weight) potently blunts the inflammatory cascades induced by MSG and effectively normalizes the cytokine milieu with reduced collagen deposition associated with fibrosis. In addition to the increased level of antioxidant enzymes, it reduces oxidative stress by inhibiting the high levels of ROS by MSG, which was in line with a previous study^17^. Quercetin can antagonize inflammatory responses, for its action is directed against several central pathways of signaling and down-regulating IL-17, IL-19, and TGF-β in sequence addition to the final point of its overall contribution to the pathogenesis of fibrosis^61,62^. Moreover, quercetin synergizes with the natural protective factors that inhibit profibrotic signaling pathways, especially the TGF-β/Smad pathway, for inhibiting EMT via reducing the vimentin level and collagen accumulation in the MSG-fed group. Additionally, histological evaluations confirmed that quercetin protects against MSG-induced fibrosis in three graded doses, but the maximum efficacy was observed in the third dose, yielding the most significant protective effects, further corroborating its role as a natural component in dysregulated cell signaling modulation. This has indicated that quercetin acts in a cellular network of interaction governing inflammation, ECM remodeling, and oxidative damage^63–65^.

**Fig. 8:**
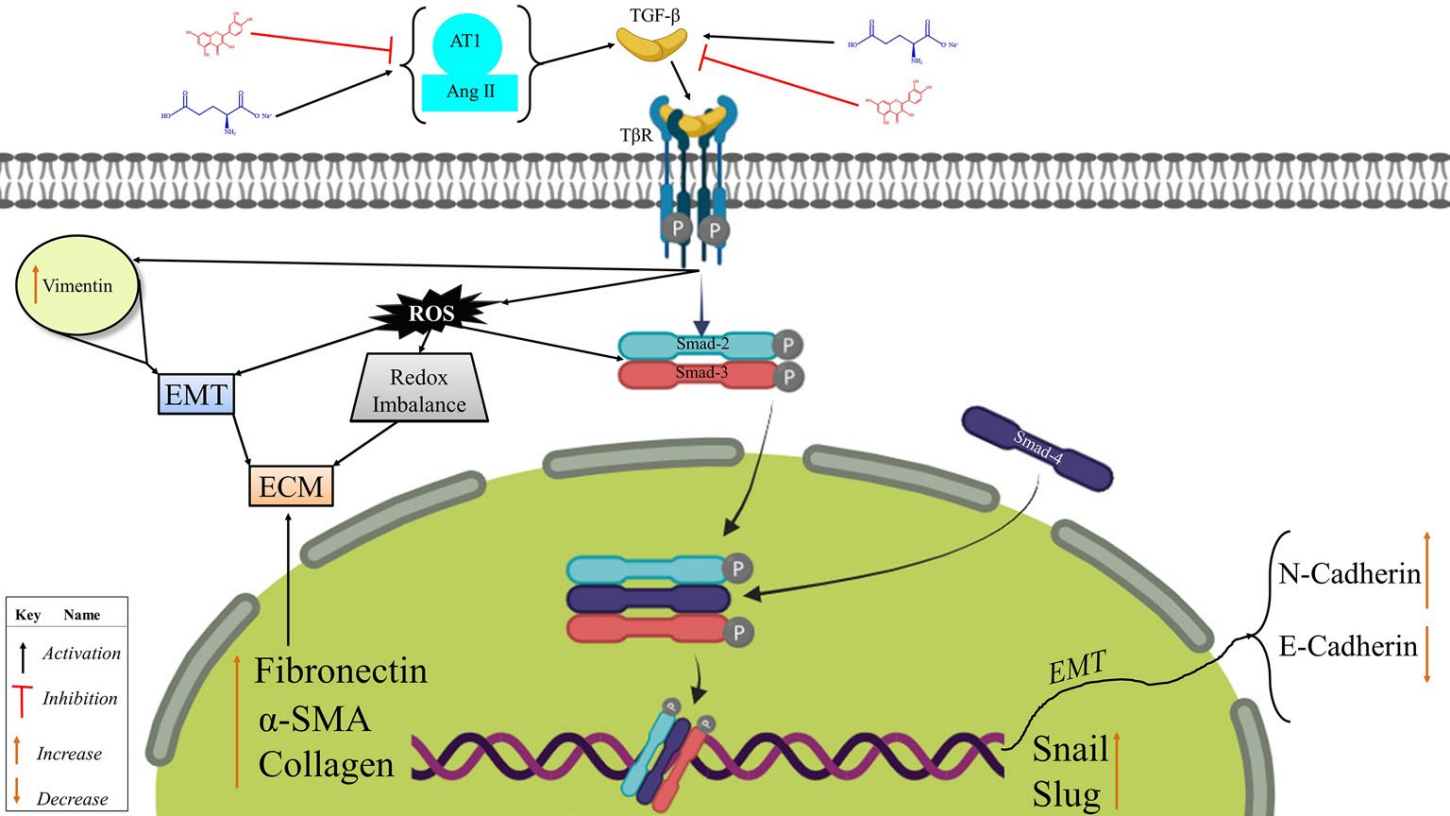
The hypothetical target pathway by which quercetin inhibits TβR and Ang II/AT1 to modulate TGF-β/Smad in MSG-induced pulmonary fibrosis (BioRender was used to make the figure: https://biorender.com).

In addition, molecular docking between MSG and quercetin presents a complementary interaction in modulating TβR-II activity coupled with its downstream Smad signaling pathway. MSG was able to display a high ability of receptor activation, as shown with a binding affinity of − 4.2 kcal/mol through which it encourages Smad signaling that contributes to fibrosis, inflammation, and other pathological processes. This is in line with scientific researches that indicate that the overexpression of TGF-β signaling leads to pathological conditions, such as fibrosis and cancer advancement, due to cell proliferation enhancement as well as extracellular matrix accumulation. Conversely, quercetin interacts more powerfully with TβR-II with an affinity of − 6.2 kcal/mol and leads to substantial conformational change when its RMSD value reaches 7.026 Å; within such conditions, receptor activation is likely affected, and thus, Smad phosphorylation will be inhibited. In many studies on pulmonary fibrosis, quercetin has been shown to inhibit the TGF-β/Smad signaling pathway, reducing markers of fibrosis and inhibiting the senescence of macrophages. Thus, in these observations, Quercetin could be regarded as an agent with therapeutic potential against the attenuation of fibrosis through the inhibition of TGF-β signaling^62,66^. Dual interaction of MSG and quercetin with TβR-II. This suggests that the signaling interaction is dynamic; hence, a combination of both positively activates the receptor in a manner that may further worsen the inflammatory and fibrotic conditions in the TβR-II. In contrast, quercetin offers an antagonistic function by inhibiting the same pathway activated by MSG. These results explain the cellular mechanisms responsible for MSG-induced lung fibrosis strongly associated with disturbances of the signaling pathways of cytokines and growth factors. The therapeutic potential of quercetin as a modulator of these pathways represents as an important advance in pharmacological intervention against MSG-induced pathologies. This finding is consistent with prior studies that showcase quercetin’s role in mitigating fibrosis and inflammation through the inhibition of TGF-β/Smad via blocking TβR and AT1 (Figs. 7E and 8). These insights suggest that quercetin could serve as a therapeutic agent in conditions where TGF-β signaling is implicated, offering a balance to the detrimental effects that might arise from compounds like MSG. Further mechanisms of action of quercetin need to be investigated to clarify which other natural compounds or even approved therapies might be synergistic with quercetin. Clinical studies are necessary to assess its *in vivo* translational potential in lung diseases to confirm its effectiveness and safety in human subjects. Unlocking the complexity of cell signaling in fibrosis and revealing any natural interventions might open up a new approach to prevent or reverse the process of fibrosis in the lung.

The central mechanism in MSG-induced pulmonary fibrosis appears to be the complex interplay of the TGF-β/Smad signaling pathway, which promotes the shift from a protective to a fibrotic state using some key inflammatory cytokines and cellular responses that are dysregulated. The present findings indicate that quercetin preserves this attenuated signaling network through its antioxidant and anti-inflammatory properties, likely because of the interference with the action of TGF-β via pathways that end in the inhibition of the deposition of collagen and decreased oxidative stress. Future research directions could focus on establishing these two aspects: the first, detailing the specific molecular ways quercetin acts with other pro-fibrotic agents, and the second, testing and potentially establishing some synergy that might occur in combination with natural supplementary compounds in the form of diet or already established therapies. More importantly, the results need to be translated into therapeutic strategies through rigorous clinical studies. This would finally do what would amount to a breakthrough in discovering the role quercetin may play in the treatment of fibrosis in the context of the use of MSG in food and evidence of effective interventions on pulmonary fibrosis.

## Electronic supplementary material

Below is the link to the electronic supplementary material.

Supplementary Material 1

## Data Availability

Data is provided within the manuscript or supplementary information files. The data underlying this research, in support of the findings, are available from the corresponding author upon reasonable request.
