# Supplementary material for: An intricate role of Ang II/AT1 in the modulation of monosodium glutamate-induced pulmonary fibrosis by TGF-β/Smad through quercetin
Source: Sci Rep. 2025 Jul 1;15:21112. doi: 10.1038/s41598-025-05781-9 (PMC12214936; doi:10.1038/s41598-025-05781-9)
Supplement: Supplementary file 1 — Supplementary Material 1 [file 41598_2025_5781_MOESM1_ESM.docx]

**Primer sequences**

| **Primer** | **Forward** | **Reverse** |
| --- | --- | --- |
| AT1^1^ | 5′-CCCACTCAAGCCTGTCTACGAA-3′ | 5′-GTGTGCTTTGAACCTGTCACTCC-3′ |
| CTGF^2^ | 5′-CGCCAACCGCAAGATTG-3′ | 5′-TACACGGACCCACCGAAGAC-3′ |
| Snai1^3^ | 5′-ACCTCCAGACCCACTCGGAT-3′ | 5′-GAGGTAGCAGGGTCAGCGAG-3′ |
| Slug^3^ | 5′-ATTCCTGGTGCGTGTCCCAT-3′ | 5′-GCAACGTGTGGGTCCGAATG-3′ |
| TGF-β1^4^ | 5′-ATTCCTGGCGTTACCTTGG-3′ | 5′-AGCCCTGTATTCCGTTCTCT-3′ |
| TβR I^5^ | 5′-TGGCGGAATCCACGAAGA-3′ | 5′-ACGGATGGATCAGAAGGTACAAG-3′ |
| TβR II^5^ | 5′-GGATGGCAAAGAGATAACCCA-3′ | 5′-AGAGTGAAGCCGTGGTAGGTGAGCTT-3′ |
| Smad 2^4^ | 5′-CGGCTGAACTGTCTCCTACC-3′ | 5′-AGGTCTCTCCAACCCTCTGG-3’ |
| Smad 3^4^ | 5′-GGTAAAGGATTGCCACCAAA-3′ | 5′-GAACAGCCAGGAAAGGGACT-3′ |
| Smad 4^6^ | 5′-CCCATCCTGGACATTACTGG-3′ | 5′-TACACCAGTCCGTCCCTTTC-3′ |
| IL-6^7^ | 5′-TTCACAAGTCCGGAGAGGAG-3′ | 5′-ACTCCAGAAGACCAGAGCAG-3′ |
| IL-1β^7^ | 5′-CAACCAACAAGTGATATTCTCCATG-3′ | 5′-GATCCACACTCTCCAGCTGCA-3′ |
| TNF-α^7^ | 5′-CTTCTGTCTACTGAACTTCG-3′ | 5′-CCAATGGCATGGATCTCAA-3′ |
| HMGB1^8^ | 5′-AGG CTG ACA AGG CTC GTT ATG-3′ | 5′-TGT CAT CCG CAG CAG TGTTG-3′ |
| MMP-2^9^ | 5′-CGTGGTGAGATCTTCTTCTTCAAGGA-3′ | 5′-CCTCATACACAGCGTCAATCTTTTC-3′ |
| MMP-9^9^ | 5′-AATTCGACTTGAAGTCTCAGAAGG-3′ | 5′-AAGTATTTGTCATGGCAGAAATAGG-3′ |
| Fibronectin^10^ | 5′-TGGCTGCCTTCAACTTCTC-3′ | 5′-AGTCCTTTAGGGCGGTCAAT-3′ |
| Col1a1^11^ | 5′-TGGCCAAGAAGACATCCCTGAAGT-3′ | 5′-ACATCAGGTTTCCACGTCTCACCA-3′ |
| Col1a2^12^ | 5′-GGAGGGAACGGTCCACGAT-3′ | 5′-GAGTCCGCGTATCCACAA-3′ |
| Col3a1^13^ | 5′-GGTTTCTTCTCACCCTGCTTC-3′ | 5′-ACAGAGGACAGATCCCGAGTC-3′ |
| α-SMA^14^ | 5′-AATGGCTCCGGGCTCTGTA-3′ | 5′-TTCCAACCATCACTCCCTGG-3′ |
| β-actin^4^ | 5′-CACCCGCGAGTACAACCTTC-3′ | 5′-CCCATACCCACCATCACACC-3′ |

**References**

1. Zhang W, Miao J, Li P, Wang Y, Zhang Y. Up-regulation of components of the renin-angiotensin system in liver fibrosis in the rat induced by CCL₄. Res Vet Sci. 2013 Aug;95(1):54-8. doi: 10.1016/j.rvsc.2013.01.028. Epub 2013 Feb 21. PMID: 23433841; PMCID: PMC7111816.
2. Chaudhary NI, Roth GJ, Hilberg F, Müller-Quernheim J, Prasse A, Zissel G, Schnapp A, Park JE. Inhibition of PDGF, VEGF and FGF signalling attenuates fibrosis. Eur Respir J. 2007 May;29(5):976-85. doi: 10.1183/09031936.00152106. Epub 2007 Feb 14. PMID: 17301095.
3. Alaaeldin R, Mohyeldin RH, Bekhit AA, Gomaa W, Zhao QL, Fathy M. Vincamine Ameliorates Epithelial-Mesenchymal Transition in Bleomycin-Induced Pulmonary Fibrosis in Rats; Targeting TGF-β/MAPK/Snai1 Pathway. Molecules. 2023 Jun 9;28(12):4665. doi: 10.3390/molecules28124665. PMID: 37375218; PMCID: PMC10303541.
4. Wang Y, Shen RW, Han B, Li Z, Xiong L, Zhang FY, Cong BB, Zhang B. Notch signaling mediated by TGF-β/Smad pathway in concanavalin A-induced liver fibrosis in rats. World J Gastroenterol. 2017 Apr 7;23(13):2330-2336. doi: 10.3748/wjg.v23.i13.2330. PMID: 28428712; PMCID: PMC5385399.
5. Mekawy DM, Sabry D, Sabry RM, Abozeid NF. Silymarin and MSC-exosomes ameliorate thioacetamide-evoked renal fibrosis by inhibiting TGF-β/SMAD pathway in rats. Mol Biol Rep. 2024 Apr 18;51(1):529. doi: 10.1007/s11033-024-09343-6. PMID: 38637422; PMCID: PMC11026270.
6. Rocha BR, Colli Sda R, Barcelos LM, Gregório BM, Sampaio FJ. Age-dependent expression of Pten and Smad4 genes in the urogenital system of Wistar rats. Acta Cir Bras. 2014;29 Suppl 1:34-8. doi: 10.1590/s0102-86502014001300007. PMID: 25185054.
7. Das D, Banerjee A, Mukherjee S, Maji BK. Quercetin inhibits NF-kB and JAK/STAT signaling via modulating TLR in thymocytes and splenocytes during MSG-induced immunotoxicity: an *in vitro* approach. Mol Biol Rep. 2024 Feb 6;51(1):277. doi: 10.1007/s11033-024-09245-7. PMID: 38319443.
8. Abdelfattah AM, Mahmoud SS, El-Wafaey DI, Abdelgeleel HM, Abdelhamid AM. Diacerein ameliorates cholestasis-induced liver fibrosis in rat via modulating HMGB1/RAGE/NF-κB/JNK pathway and endoplasmic reticulum stress. Sci Rep. 2023 Jul 15;13(1):11455. doi: 10.1038/s41598-023-38375-4. PMID: 37454204; PMCID: PMC10349817.
9. Aouey B, Boukholda K, Ciobica A, Burlui V, Soulimani R, Chigr F, Fetoui H. Renal Fibrosis and Oxidative Stress Induced by Silica Nanoparticles in Male Rats and Its Molecular Mechanisms. Iran J Pharm Res. 2024 Mar 26;23(1):e143703. doi: 10.5812/ijpr-143703. PMID: 38655071; PMCID: PMC11036645.
10. Abd-Elhakim YM, Moustafa GG, Hashem MM, Ali HA, Abo-El-Sooud K, El-Metwally AE. Influence of the long-term exposure to tartrazine and chlorophyll on the fibrogenic signalling pathway in liver and kidney of rats: the expression patterns of collagen 1-α, TGFβ-1, fibronectin, and caspase-3 genes. Environ Sci Pollut Res Int. 2019 Apr;26(12):12368-12378. doi: 10.1007/s11356-019-04734-w. Epub 2019 Mar 7. PMID: 30847814.
11. da Silva AF, Silva K, Reis LA, Teixeira VP, Schor N. Bone Marrow-Derived Mesenchymal Stem Cells and Their Conditioned Medium Attenuate Fibrosis in an Irreversible Model of Unilateral Ureteral Obstruction. Cell Transplant. 2015;24(12):2657-66. doi: 10.3727/096368915X687534. Epub 2015 Feb 18. PMID: 25695732.
12. Zhang GB, Song YN, Chen QL, Dong S, Lu YY, Su MY, Liu P, Su SB. Actions of Huangqi decoction against rat liver fibrosis: a gene expression profiling analysis. Chin Med. 2015 Dec 18;10:39. doi: 10.1186/s13020-015-0066-5. PMID: 26691002; PMCID: PMC4683959.
13. Wang J, Li Z, Du J, Li J, Zhang Y, Liu J, Hou Y. The expression profile analysis of atrial mRNA in rats with atrial fibrillation: the role of IGF1 in atrial fibrosis. BMC Cardiovasc Disord. 2019 Feb 15;19(1):40. doi: 10.1186/s12872-019-1013-7. PMID: 30770724; PMCID: PMC6377759.
14. Su DN, Wu SP, Xu SZ. Mesenchymal stem cell-based Smad7 gene therapy for experimental liver cirrhosis. Stem Cell Res Ther. 2020 Sep 14;11(1):395. doi: 10.1186/s13287-020-01911-4. PMID: 32928296; PMCID: PMC7489041.
